# Enhancing Robotic Surgery Training and Reducing Remote Complications with Telesurgery Technology

**DOI:** 10.1590/S1677-5538.IBJU.2025.0082

**Published:** 2025-03-20

**Authors:** Marcio Covas Moschovas, Shady Saikali, Travis Rogers, Mischa Dohler, Michael Mcdonald, Ela Patel, Jeffrey Marquinez, Ahmed Gamal, Jeffery Magnuson, Vipul Patel

**Affiliations:** 1 AdventHealth Global Robotics Institute Kissimmee FL USA AdventHealth Global Robotics Institute, Kissimmee, FL, USA; 2 University of Central Florida Orlando FL USA University of Central Florida - UCF, Orlando, FL, USA; 3 Advanced Technology Group, Ericsson Inc. Santa Clara USA Advanced Technology Group, Ericsson Inc., Santa Clara, USA

## Abstract

**Introduction:**

The 2001 Lindbergh operation provided evidence for the feasibility of transatlantic telesurgery.(1-3) However, technological and economic challenges have limited the implementation of this technique.(4-6) This video illustrates details of a telesurgery connection over a 13,000 km distance between Orlando (USA) and Shanghai (China). Surgeons at both locations operated simultaneously on the same animals using telesurgery consoles (MicroPort® MedBot™) for teleproctoring, allowing for a robust evaluation of connectivity and robotic system performance across vast distances.

**Methods:**

On July 23rd and 24th, 2024, we conducted a prospective telesurgery study using live animal models (porcine) connecting Orlando to Shanghai. We reproduced a real-life telesurgery scenario where both ends of the connection had control over the robot. Four surgeons were in Orlando and one in Shanghai. We illustrated the communication between surgeons and highlighted the potential of telesurgery to improve outcomes and teaching robotic surgery.

**Results:**

Connectivity and robotic technology performed optimally for several hours without troubleshooting or malfunctions. Median delay was 139 milliseconds (137-216) on the first day and 139 milliseconds (137-185) on the second day. The surgeons were able to switch the console control multiple times during the procedures. They could communicate, discuss cases in real-time, and seamlessly transfer control in critical steps of the surgery.

**Conclusions:**

This video underscores the practical potential of Telesurgery use in teleproctoring, particularly when an experienced remote surgeon steps in to assist another surgeon during complex or challenging procedures. It highlights Telesurgery's potential for training and improving outcomes in robotic surgery.

Available at: http://www.intbrazjurol.com.br/video-section/20250082_Moschovas_et_al
